# Supplementary material for: Catalytic Conversion of Xylose to Furfural by p-Toluenesulfonic Acid (pTSA) and Chlorides: Process Optimization and Kinetic Modeling
Source: Molecules. 2021 Apr 12;26(8):2208. doi: 10.3390/molecules26082208 (PMC8070381; doi:10.3390/molecules26082208)
Supplement: Supplementary file 1 [file molecules-26-02208-s001.pdf]

Catalytic conversion of xylose to furfural by *p*-toluenesulfonic acid (*p*TSA) and chlorides: Process optimization and kinetic modeling

Muhammad Sajid[1,2,3,*], Muhammad Rizwan Dilshad[4], Muhammad Saif Ur Rehman[5], Dehua Liu[2], Xuebing Zhao [2,*]

[1] *Faculty of Materials and Chemical Engineering, Yibin University, Yibin 644000, Sichuan China;*

[2]*Institute of Applied Chemistry, Department of Chemical Engineering, Tsinghua University, Beijing 100084, China*

[3]*Department of Chemical Engineering, University of Gujrat, Gujrat 50700 Pakistan;*

[4]*Institute of Chemical Engineering and Technology, University of the Punjab, Lahore, Pakistan*

[5]*Department of Chemical Engineering, Khwaja Fareed University of Engineering and Information Technology, Abu Dhabi Road, Rahim Yar Khan, Pakistan*

* Corresponding author: MS engr.sajid80@gmail.com Tel: +86 17810264474 ORCID: 0000-0001-9471-8395;

XZ zhaoxb@mail.tsinghua.edu.cn

Scheme S1. Reaction mechanism of acid catalyzed dehydration of xylose to furfural[1,2]

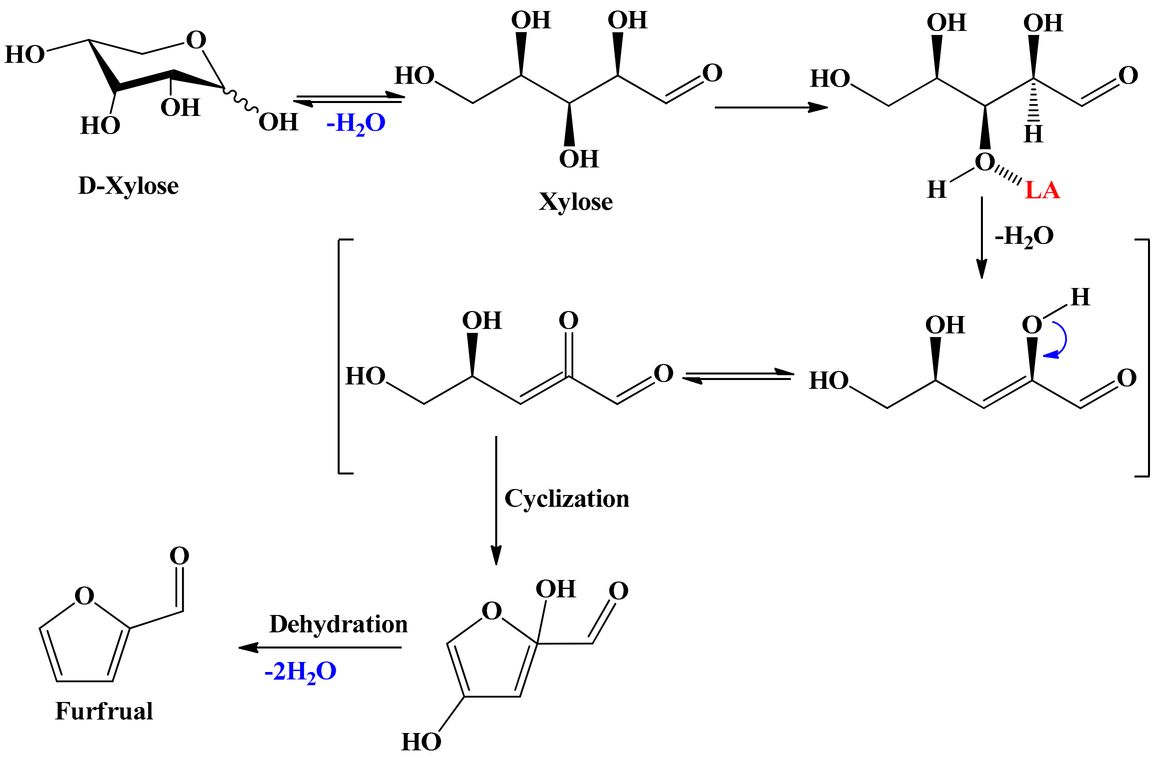

Table S1. Xylose transformation into furfural. Reaction conditions: 1.0 M xylose with 1.0 M organic acid in 50 ml water solvent medium heated in an oil bath at 100 ºC and stirred at 200 rpm for 8 hours.

**Organic acid**	**Xylose conversion (%)**	**Furfural yield (%)**
*p*TSA	24.2±2.1	2.91
Oxalic acid	20.5±2.0	<1
Maleic acid	12.7±1.5	<1
Malonic acid	11.7±3.0	<1
Succinic acid	6.4±3.5	<1

Table S2. Fitted kinetic constants of organic acid catalyzed conversion of xylose into furfural in the water medium. Reaction conditions: 0.5 M Xylose with 1.0 M *p*TSA and 0.1 M Lewis acid catalyst in 50 ml water heated in an oil bath at 100 ºC with a stirring rate of 200 rpm for 8 hours.

**Lewis acid**	**Rate constant (min^{-1})**			**Goodness of Fit (R^2)**		**Max yield**
	k_{1obs}	k_{2obs}	k_{3obs}	**Xylose**	**Furfural**	Y_{max}
No Lewis acid (only *p*TSA)	6.08×10^{-5}	7.17×10^{-4}	1.02×10^{-11}	0.8505	0.9091	2.61±2.5
NH_4Cl	2.01×10^{-4}	5.12×10^{-4}	2.22×10^{-14}	0.955	0.9926	8.3±2.5
$CrCl_3.6H_2O$	5.58×10^{-4}	1.18×10^{-3}	1.16×10^{-9}	0.950	0.9905	17.6±2.4
$AlCl_3$	2.20×10^{-4}	4.88×10^{-4}	2.22×10^{-14}	0.8476	0.9947	8.8±2.2

Figure S1. Kinetic plots of *p*TSA-catalyzed conversion of xylose into furfural in the water medium. Reaction conditions: 0.5 M xylose with 1.0 M *p*TSA and 0.1 M Lewis acid catalyst in 50 ml deionized water heated in oil bath at 100 ^{o}C for 8 h with a stirring speed of 200 rpm; (A) No Lewis acid addition (only *p*TSA); (B) NH_4Cl; (C) $CrCl_3.6H_2O$; (D) $AlCl_3$. Lines are for model-predicted data, and symbols are for experiment-determined data.

X: xylose; FF: furfural; HUM; humin

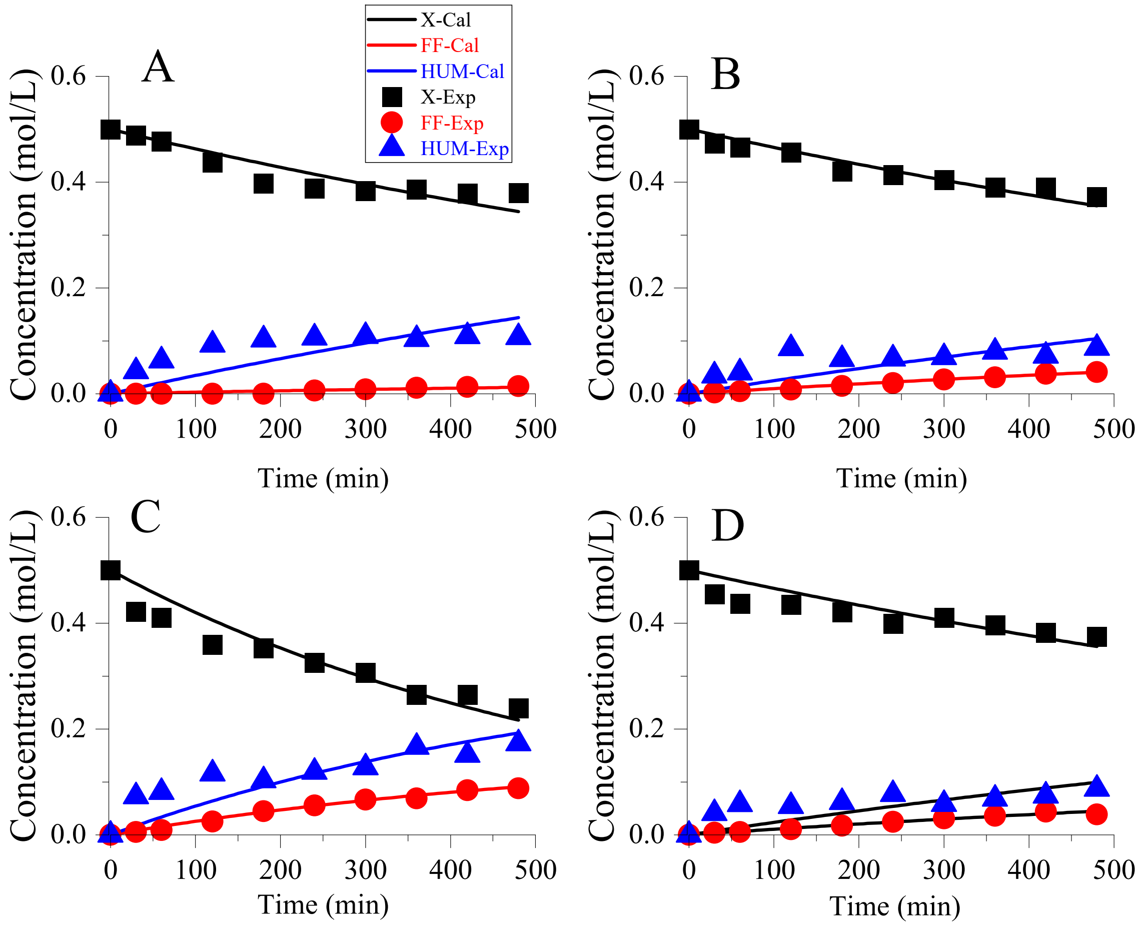

Table S3. Fitted kinetic constants of organic acid catalyzed conversion of xylose into furfural in the water medium. Reaction conditions: 0.5 M Xylose with 1.0 M *p*TSA and 0.1 – 0.6 M $CrCl_3.6H_2O$ catalyst in 50 ml water at 100 ºC with a stirring rate of 200 rpm for 8 h.

**$CrCl_3.6H_2O$ Conc. (M)**	**Rate constant (min^{-1})**			**Goodness of Fit (R^2)**		**Max yield**
	k_{1obs}	k_{2obs}	k_{3obs}	**Xylose**	**Furfural**	Y_{max}
0.1	5.58×10^{-4}	1.18×10^{-3}	1.16×10^{-9}	0.95	0.9905	17.6±1.2
0.2	7.98×10^{-4}	1.35×10^{-3}	2.76×10^{-10}	0.9874	0.9922	23.4±1.1
0.3	1.23×10^{-3}	2.17×10^{-3}	4.79×10^{-12}	0.9755	0.9755	26.6±1.5
0.4	1.16×10^{-3}	1.98×10^{-3}	1.14×10^{-4}	0.9744	0.981	26.8±1.4
0.5	1.70×10^{-3}	2.97×10^{-3}	5.21×10^{-11}	0.9744	0.9855	30.1±0.9
0.6	2.02×10^{-3}	3.24×10^{-3}	4.28×10^{-4}	0.9659	0.9769	29.5±1.1

Figure S2. Kinetic plots of *p*TSA-catalyzed conversion of xylose into furfural in water solvent medium. Reaction conditions: 0.5 M xylose with 1.0 M *p*TSA and 0.1 – 0.6 M $CrCl_3.6H_2O$ catalyst in 50 ml water heated in oil bath at 100 ºC for 8 h with a stirring speed of 200 rpm; (A) 0.1 M $CrCl_3.6H_2O$; (B) 0.2 M $CrCl_3.6H_2O$; (C) 0.3 M $CrCl_3.6H_2O$; (D) 0.4 M $CrCl_3.6H_2O$; (E) 0.5 M $CrCl_3.6H_2O$; (F) 0.6 M $CrCl_3$. Lines are for model-predicted data, and symbols are for experiment-determined data. X: xylose; FF: furfural; HUM; humin

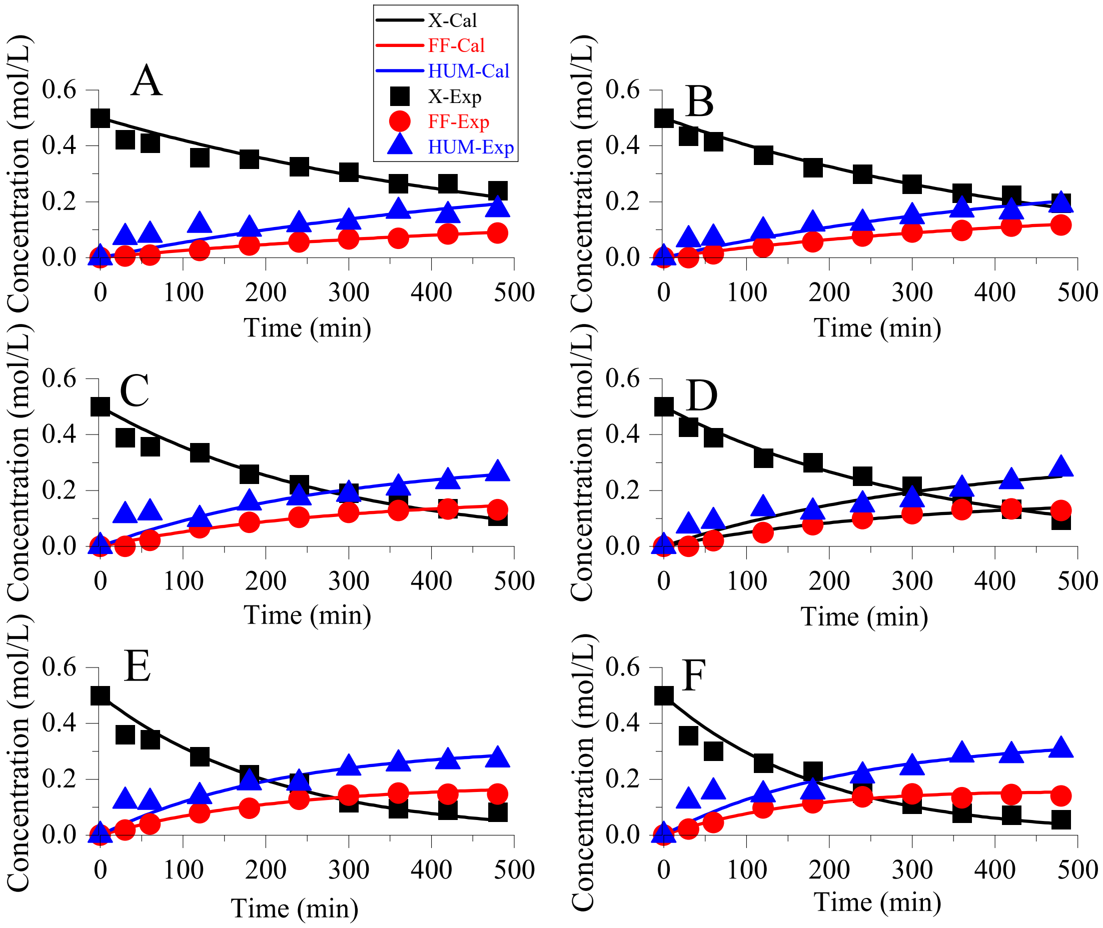

Figure S3. Kinetic plots of *p*TSA-catalyzed conversion of xylose into furfural in the DMSO solvent medium. Reaction conditions: 0.5 M xylose with 1.0 M *p*TSA and 0.5 M $CrCl_3.6H_2O$ catalysts in 50 ml DMSO heated in oil bath for 5 h with a stirring speed of 200 rpm; the flask was heated at; (A) 110 ºC; (B) 120 ºC; (C) 130 ºC; (D) 140 ºC; (E) 150 ºC; (F) 160 ºC. Lines are for model-predicted data, and symbols are for experiment-determined data. X: xylose; FF: furfural; HUM; humin

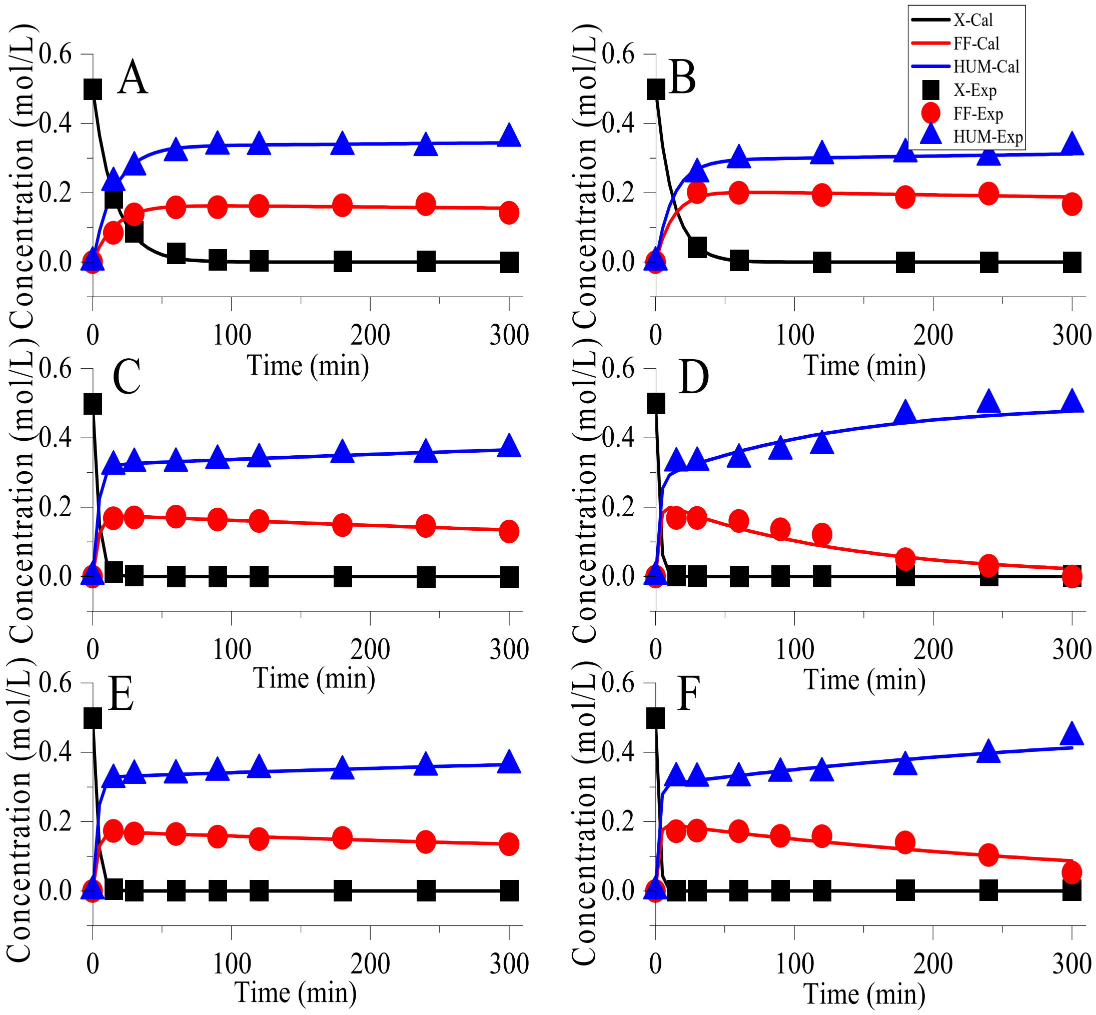

Figure S4. Kinetic plots of *p*TSA-catalyzed conversion of xylose into furfural in the DMSO solvent medium. Reaction conditions: 0.5 M xylose with 1.0 M *p*TSA and $CrCl_3.6H_2O$ catalysts in 50 ml DMSO heated in oil bath for 5 h with a stirring speed of 200 rpm; the flask was heated in oil bath at 120 ºC using different concentrations of $CrCl_3.6H_2O$; (A) 0.1 M; (B) 0.2 M; (C) 0.3 M; (D) 0.4 M; (E) 0.5 M. Lines are for model-predicted data, and symbols are for experiment-determined data. X: xylose; FF: furfural; HUM; humin

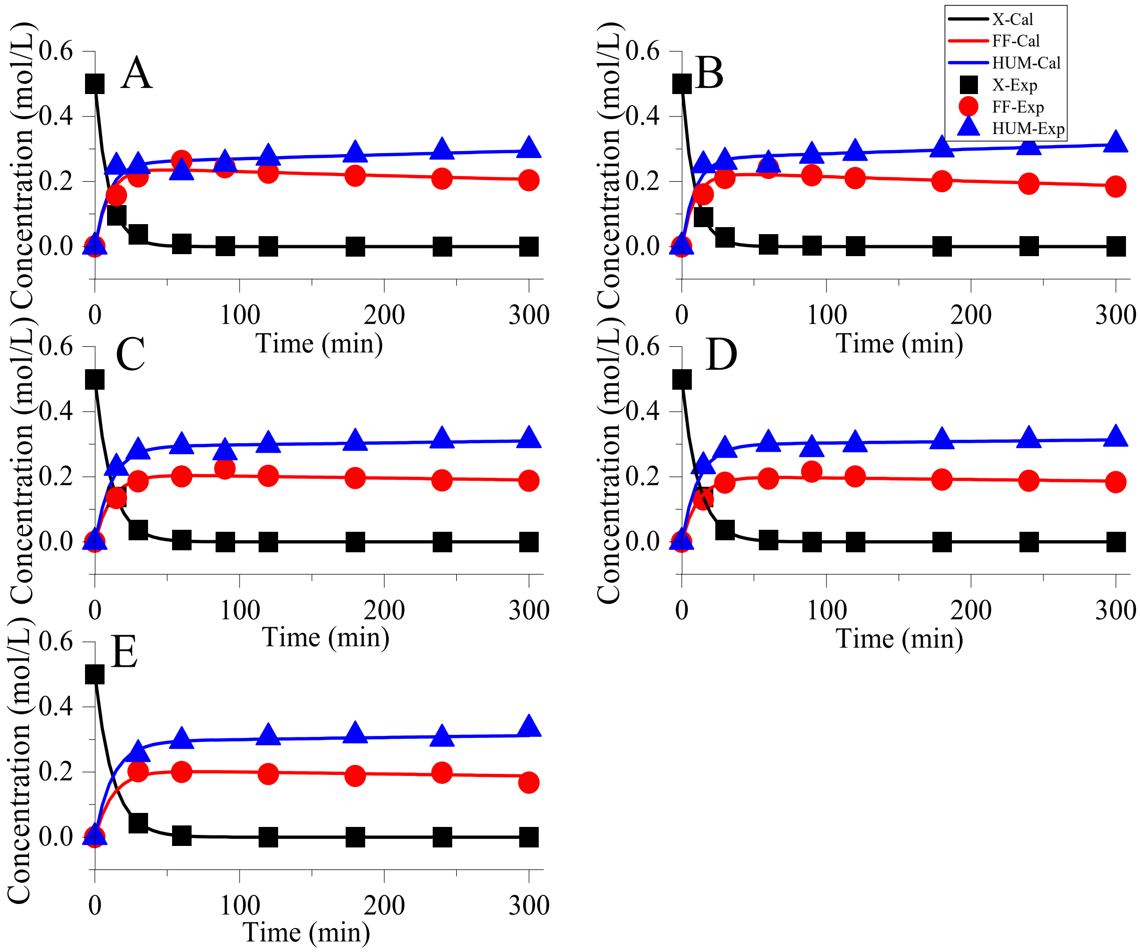

Table S4. Fitted kinetic constants of organic acid catalyzed conversion of xylose into furfural in DMSO solvent medium. Reaction conditions: 0.25 – 1.0 M Xylose with 1.0 M *p*TSA and 0.1 M $CrCl_3.6H_2O$ catalysts in 50 ml DMSO with a stirring rate of 200 rpm heated in an oil bath at 120 °C for 5 h.

Xylose (M)	Rate constant (min^{-1})			Goodness of Fit (R^2)			Max yield	t_{Ymax} (h)
	k_{1obs}	k_{2obs}	k_{3obs}	Xylose	Furfural	Humin	Y_{max}	
0.25	0.0473	0.0471	7.76×10^{-4}	0.9999	0.956	0.9641	53.1±1.9	1
0.5	0.0508	0.0542	5.49×10^{-4}	0.9990	0.9543	0.9538	52.60±1.1	1
1.0	0.0312	0.0434	6.89×10^{-4}	0.9990	0.9327	0.9551	42.4±2.0	1.5

Figure S5. Kinetic plots of organic acid-catalyzed conversion of xylose into furfural in the DMSO solvent medium. Reaction conditions: 0.25 – 0.5 M xylose with 1.0 M organic acid and 0.1 M $CrCl_3.6H_2O$ catalysts in 50 ml DMSO heated in an oil bath at 120 ºC for 5 h with a stirring speed of 200 rpm; (A) 0.25 M; (B) 0.5 M ; (C) 1.0 M. Lines are for model-predicted data, and symbols are for experiment-determined data. X: xylose; FF: furfural; HUM; humin

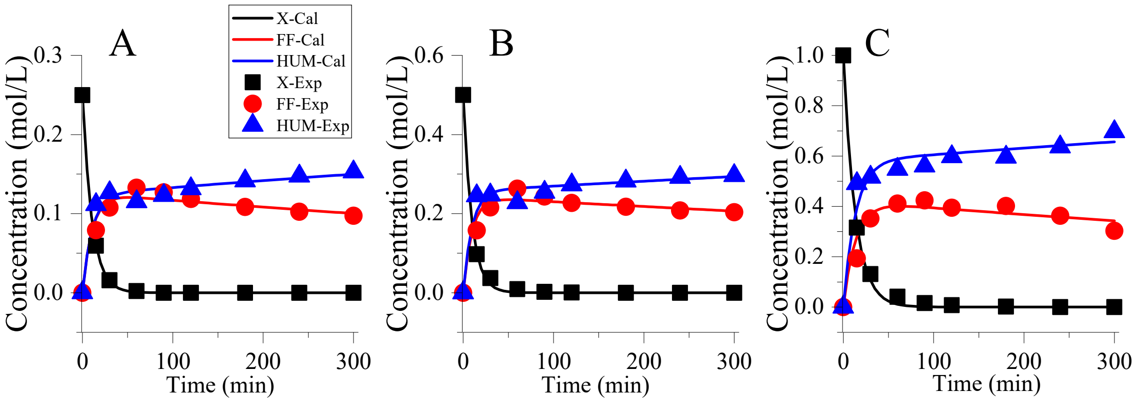

Table S5. Comparison of the activation energy for acid-catalyzed conversion of xylose to furfural in different solvent systems

Catalyst	Solvent	Temperature (^{o}C)	Activation Energy (kJ/mol)		Ref.,
			k_{1obs}	k_{2obs}	
pTSA-$CrCl_3.6H_2O$	DMSO	110 - 160	81.8	66.5	This study
Formic acid	Water	130 - 200	152	161	[2]
Acetic acid	Water	170 - 210	108.6	105	[3]
ZSM-5 zeolite	Water	140 – 220<br>10 MPa	134.2	-	[4]
non-catalyzed	Water<br>(HTLW)	180 – 220<br>10 MPa	111.5	143.1	[5]
non-catalyzed	Water<br>(HTLW)	140 – 240<br>3 MPa	76.6	153.8/<br>58.8[a]	[6]
H_2SO_4	Water	155 - 185	82.8		[7]
HCl	MIBK-water	140 -170	123.9	72.5	[8]
HCl-NaCl	Water	160 – 200	133.3	125.8	[9]
H_2SO_4	Water	145 - 175	145	-	[10]
H_2SO_4	GVL	145 - 175	114	-	

[a] The two values represent a combined first- and second-order reaction, respectively.

Bibliography

1. Gupta, N.K.; Fukuoka, A.; Nakajima, K. Amorphous Nb2O5 as a Selective and Reusable Catalyst for Furfural Production from Xylose in Biphasic Water and Toluene. *ACS Catal.* **2017**, *7*, 2430–2436, doi:10.1021/acscatal.6b03682.

2. Lamminpää, K.; Ahola, J.; Tanskanen, J.; Lamminpä, K.; Ahola, J.; Tanskanen, J. Kinetics of Xylose Dehydration into Furfural in Formic Acid. *I EC Res.* **2012**, *51*, 6297–6303, doi:10.1021/ie2018367.

3. Chen, Z.; Zhang, W.; Xu, J.; Li, P. Kinetics of xylose dehydration into furfural in acetic acid. *Chinese J. Chem. Eng.* **2015**, *23*, 659–666, doi:10.1016/j.cjche.2013.08.003.

4. O'Neil, R.; Ahmad, M.N.; Vanoye, L.; Aiouache, F. Kinetics of aqueous phase dehydration of xylose into furfural catalyzed by ZSM-5 zeolite. *Ind. Eng. Chem. Res.* **2009**, *48*, 4300–4306, doi:10.1021/ie801599k.

5. Jing, Q.; Lu, X.; Yuan, L. Kinetics of non-catalyzed decomposition of xylose in high temperature liquid water. *Huaxue Fanying Gongcheng Yu Gongyi/Chemical React. Eng. Technol.* **2006**, *22*, 472–475, doi:10.1016/s1004-9541(07)60143-8.

6. Kim, S.B.; Lee, M.R.; Park, E.D.; Lee, S.M.; Lee, H.; Park, K.H.; Park, M.J. Kinetic study of the dehydration of d-xylose in high temperature water. *React. Kinet. Mech. Catal.* **2011**, *103*, 267–277, doi:10.1007/s11144-011-0320-5.

7. Shi, S.; Guan, W.; Kang, L.; Lee, Y.Y. Reaction kinetic model of dilute acid-catalyzed hemicellulose hydrolysis of corn stover under high-solid conditions. *Ind. Eng. Chem. Res.* **2017**, *56*, 10990–10997, doi:10.1021/acs.iecr.7b01768.

8. Weingarten, R.; Cho, J.; Conner, Jr., W.C.; Huber, G.W.; Conner, W.C.; Huber, G.W. Kinetics of furfural production by dehydration of xylose in a biphasic reactor with microwave heating. *Green Chem.* **2010**, *12*, 1423–1429, doi:10.1039/c003459b.

9. Danon, B.; Hongsiri, W.; van der Aa, L.; de Jong, W. Kinetic study on homogeneously catalyzed xylose dehydration to furfural in the presence of arabinose and glucose. *Biomass and Bioenergy* **2014**, *66*, 364–370, doi:10.1016/j.biombioe.2014.04.007.

10. Mellmer, M.A.; Sener, C.; Gallo, J.M.R.; Luterbacher, J.S.; Alonso, D.M.; Dumesic, J.A. Solvent effects in acid-catalyzed biomass conversion reactions. *Angew. Chemie - Int. Ed.* **2014**, *53*, 11872–11875, doi:10.1002/anie.201408359.
